# Expression profiling of key pathways in rat liver after a one-year feeding trial with transgenic maize MON810

**DOI:** 10.1038/s41598-019-55375-5

**Published:** 2019-12-12

**Authors:** Torsten Stein, Guangyao Ran, Marc Bohmer, Soroush Sharbati, Ralf Einspanier

**Affiliations:** 1 Institute of Veterinary Biochemistry, Freie Universität Berlin, Oertzenweg 19b, 14163 Berlin, Germany; 2 Present Address: Department of Liquor Making Engineering, Moutai Institute, Luban Avenue, 564507 Renhuai, China; 3 Present Address: SGS Institute Fresenius GmbH, Life Sciences Services, Tegeler Weg 33, 10589 Berlin, Germany

**Keywords:** Gene expression, Transcriptomics

## Abstract

In a recent one-year feeding study, we observed no adverse effects on tissue level in organs of rats fed with the genetically-modified maize MON810. Here, we assessed RNA expression levels of 86 key genes of the apoptosis-, NF-кB-, DNA-damage response (DDR)-, and unfolded-protein response (UPR) pathways by RT-qPCR in the rat liver. Male and female rats were fed either with 33% MON810 (GMO), isogenic- (ISO), or conventional maize (CONV) and RNAs were quantified from eight rats from each of the six feeding groups. Only *Birc2* transcript showed a significant (p ≤ 0.05) consistent difference of ≥1.5-fold between the GMO and ISO groups in both sexes. Unsupervised cluster analysis showed a strong separation of male and female rats, but no clustering of the feeding groups. Individual analysis of the pathways did not show any clustering of the male or female feeding groups either, though transcript levels of UPR pathway-associated genes caused some clustering of the male GMO and CONV feeding group samples. These differences were not seen between the GMO and ISO control or within the female cohort. Our data therefore does not support an adverse effect on rat liver RNA expression through the long-term feeding of MON810 compared to isogenic control maize.

## Introduction

Maize is one of the most important cereal crops for humans and animals with over 800 million tons of maize grown on 148 million hectares worldwide^1^. Transgenic maize has been cultivated since 1996 with more than 50 million hectares of genetically modified (GMO) maize planted in 2015 alone^2^, and in the USA GMO hybrid varieties made up ~90% of the maize grown^1^. One of the most widespread transgenic maize varieties, MON810, expresses the pesticidal crystal protein Cry1Ab from Bacillus thuringiensis (Bt), which is virulent against the European corn borer but is understood to have no adverse effect on higher mammals. Damage through this pest has been significant with a reduction in yields of up to 5%. Resistance of Bt-maize against the corn borer therefore can potentially lead to increased crop yield and reduced use of insecticides. However, safety concerns of such genetically modified crops are still an ongoing worldwide debate.

Over the last years, numerous research activities have focused on the safety assessment of such genetically modified crops, using various animal models, including mouse^3^, rat^4–7^, cattle^8^, salmon^9^, poultry^7,10^ and pig^11,12^.

Systematic evaluation of potential side effects of Bt-maize versus conventional maize have been carried out to assess any significant differences using animal feeding studies. These studies concluded that the Bt-maize was as safe and nutritious as existing conventional corn varieties^4,13,14^. However, these results were questioned by the Séralini lab^15^ who after reanalysis of Monsanto’s data announced that the results of the three GMO studies were sex- and often dose-dependent and showed side effects associated with the kidney and liver, although these were not consistent between the three studies. Effects on the heart, adrenal glands, spleen and haematopoietic system were further described by this group^15^. Additionally, an *in vitro* study in which the human embryonic kidney 293 cell line was treated with increasing amounts of Cry1Ab and Cry1Ac protein found that 100 ppm Cry1Ab caused cell death with no effects by Cry1Ac under the experimental conditions^16^. Therefore, there is still a need to evaluate the potential risk of Cry1Ab in GMO maize for the consuming animal.

Even though a large number of studies have assessed the potential risk of GMO, very few studies have used the necessary mid- to long-term feeding studies with GMO maize. To our knowledge, only four studies in mice^17^, rats^14,18^ and sheep^19^ have been conducted to test the long-term effect of feeding with GM crops compared to conventional crops but no year long-term feeding study using MON810 maize had been carried out. Our own group previously presented data of a 90-day feeding study from rats that received diets containing either 33% GM maize (MON810) or near-isogenic control maize, in which no biological response to the GM-diet was observed in either male or female rat intestinal tissues^20^. In contrast, de Vendomois *et al*.^15^ re-analysed data from a 90-day feeding study using, a Cry3Bb1-expressing variety, first described by Monsanto^21^ as well as MON810 and NK 603, which is tolerant to the broad spectrum herbicide ‘Roundup’. The reanalysis identified a sex-dependent slight variation in body weight and signs of hepatorenal toxicity in rats, therefore demanding further long-term experiments. Such long-term studies are critical as short-term studies can potentially show effects that may not manifest themselves long-term.

As part of the European Commission-funded GRACE project (GMO Risk Assessment and Communication of Evidence; www.grace-fp7.eu) within the 7th Framework Programme we therefore recently carried out a 1-year feeding study in which male and female Wistar Han RCC rats were fed either a MON810-variety, its near-isogenic non-GM comparator, or an additional conventional maize variety. This work was done according to the guidelines by the EFSA Scientific Committee in 2011 and the OECD Test Guideline 452. These results showed that the MON810 maize at a level of up to 33% in the diet did not induce adverse effects in either male or female rats regarding body weight, relative organ weight, haematology, clinical biochemistry or differential leukocyte count^22^.

We have now followed up this study by characterising the liver transcript profiles of key cellular pathways during the one-year MON810 feeding regime compared to isogenic non-GM maize and conventional maize to identify any possible gene expression changes not manifested on morphological level. Given the results by de Vendomois *et al*.^15^ mentioned above, we focussed our analysis on the rat liver. Expression of RNA coding for proteins of the apoptosis-, NF-kB-, DNA damage response- (DDR), and the unfolded-protein response (UPR) pathways were investigated by RT-qPCR on total RNA and partly by Western blot.

## Results

### qPCR analysis of genes related to key cellular pathways does not detect major transcriptional changes by a one-year MON810-feeding regime

To assess possible RNA expression changes in the liver of MON810-fed rats, 86 transcripts related to key pathways were assessed by RT-qPCR overall. These marker genes included 26 genes coding for proteins associated with apoptosis, 24 genes of unfolded protein response pathways, 15 genes of the NF-кB pathway and 21 genes of the DNA damage and repair (DDR) pathways. We randomly selected 10 rats per group for RNA isolation and chose eight extracts from each group that fulfilled all the necessary quality requirements for further analysis, so that a total of 48 rat livers were assessed for each RNA. Average ΔCt values were calculated by normalising to the average expression Ct values of three housekeeping genes.

81/86 RNAs were below our detection cut-off (ΔCt < 9) in both male and females. ΔCt values for these RNAs were then compared between the MON810- (GMO), isogenic control- (ISO) and conventional maize control (CONV)-fed rat cohorts to obtain ΔΔCt values for each RNA, and presented as fold-change through −2^ΔΔCt^ (see Table S2 for full results). Male and female cohorts were analysed separately to be able to identify any possible sex-dependent changes. Statistically significant changes were defined as p ≤ 0.05.

**Figure 1:**
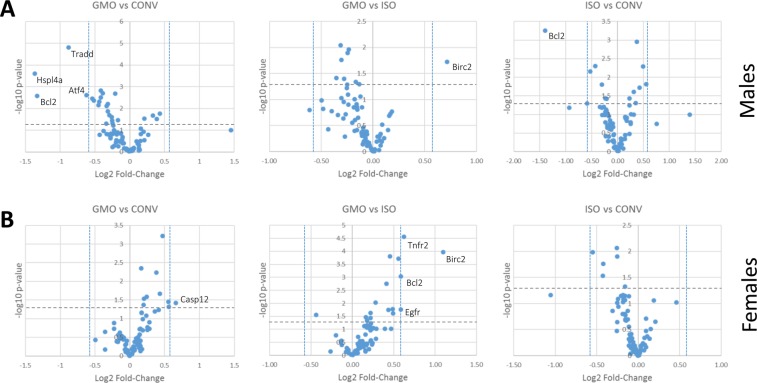
Comparison of RNA abundances of the individual feeding groups within the male and female cohorts. Average ΔCt values of 81 RNAs detected in isolates from the liver of the GMO-, ISO-, and CONV-fed rats were plotted as volcano plots in which the negative log10 of the p-values was plotted against the log2 of the fold-change. The horizontal dashed line shows the p ≤ 0.05 cut-off, while the two vertical dotted lines represent the ≥1.5-fold cut-off. Male (**A**) or female (**B**) cohorts were analysed separately. Significantly changed RNAs of ≥1.5-fold change (left and right hand top corners) have been highlighted.

In the male cohort, GMO vs ISO comparison revealed eight genes with statistically significant changes (1 up, 7 down) of which only one was changed ≥1.5-fold (*Birc2*) (Fig. 1A). This low arbitrary cut-off was chosen to include very small changes. The GMO vs CONV comparison showed 25 RNAs with statistically significant changes (4 up, 21 down) of which only four genes (*Atf4, Bcl2, Hspl4A,Tradd*) changed ≥1.5-fold (all down) and only *Hspl4A* > 2-fold. However, the ISO vs CONV comparison also showed 13 statistically significant changes (7 up, 6 down) of which one (*Bcl2*) also decreased ≥1.5-fold (Fig. 1A; Table S2).

In the female cohort, GMO vs ISO comparison revealed 16 genes with statistically significant changes (15 up, 1 down) of which four changed ≥1.5-fold (*Bcl2, Birc2, Egfr, Tnfr2*; all up) and only *Birc2* > 2-fold (Fig. 1B). The GMO vs CONV comparison showed 10 statistically significant changes (all up) of which only *Casp12* changed ≥1.5-fold. The ISO vs CONV comparison also showed six statistically significant changes (all down), none of which changed ≥1.5-fold (Fig. 1B; Table S2).

**Figure 2:**
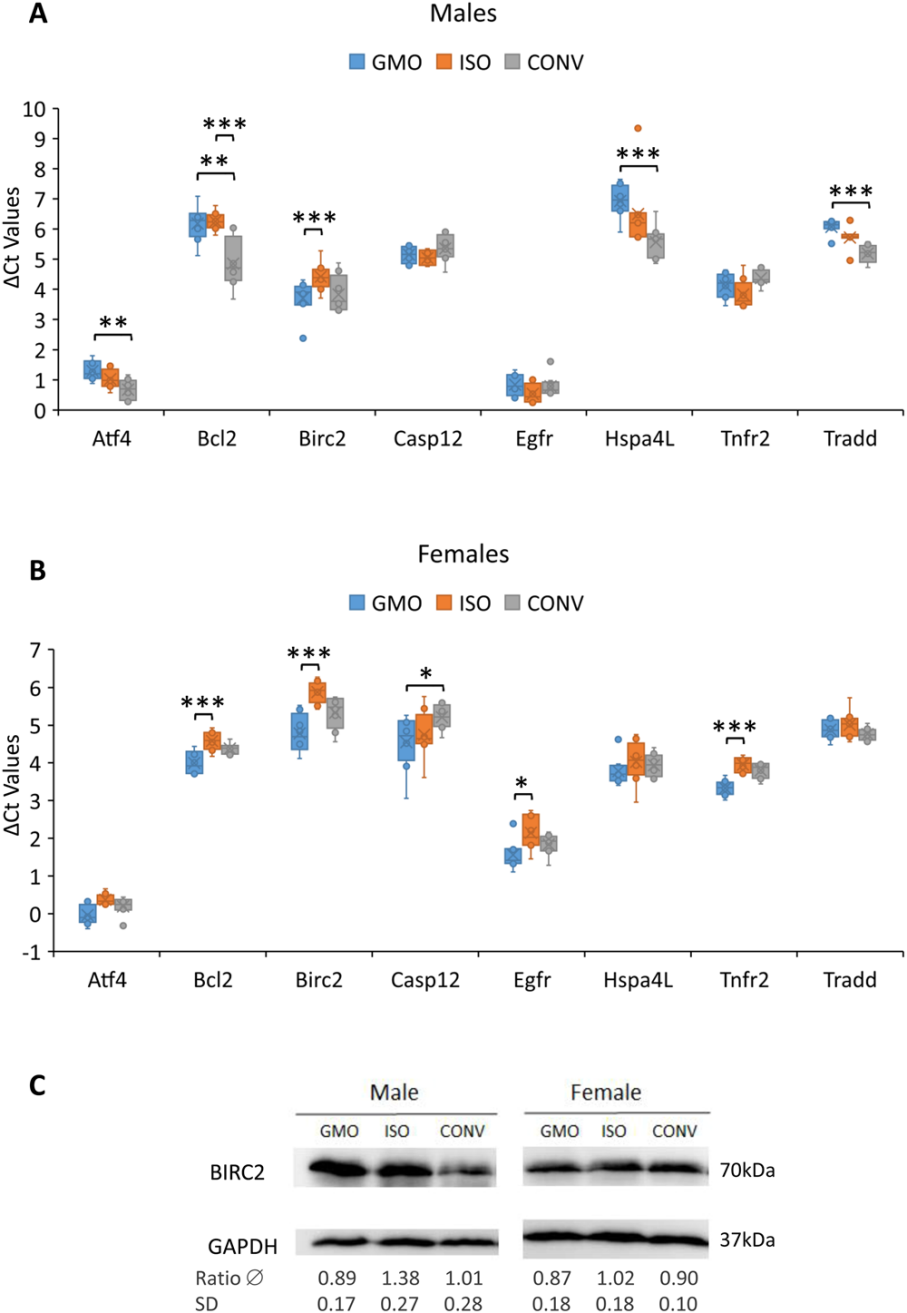
Comparison of abundance of RNAs with statistically significant changes (≥1.5-fold) between any of the three feeding groups. Box and Whisker plots of the ΔCt values show the statistically significant changes of ≥1.5-fold between the different feeding groups (GMO, ISO, CONV) in either the male (**A**) or female (**B**) cohort, as well as the variations of ΔCt values for each RNA (bars) and individual outliers (dots). ***p ≤ 0.001; **p ≤ 0.01; *p ≤ 0.05. (**C**) Semi-quantitative detection of BIRC2 protein. Western blot depicting examples for BIRC2 protein within liver extracts of male and female rats fed GMO, isogenic or conventional maize. Mean BIRC2/GAPDH ratios (Ratio ∅) have been obtained from three independent Western blots by densitometry and are shown with standard deviation (SD).

Overall, only one RNA, *Birc2*, showed a significant difference in abundance between the GMO and ISO groups above our 1.5-fold cut-off in both the male and female cohort. However, this RNA was not significantly changed ≥1.5-fold in the GMO vs CONV comparisons (Fig. 2A,B). The only other RNAs with a statistically significant change in the GMO vs ISO comparison in both sexes were *Fadd* and *Traf2*, though this was well below our 1.5-fold cut-off and changed in opposite directions within male and female rat cohorts. Similar significant changes for *Traf2*, however, were also seen in the ISO vs CONV comparison for this RNA (Table S1).

To test whether the up-regulation of *Birc2* mRNA was also translated into an increase on protein level, a Western blot was performed using pooled protein extracts from all eight male and female rats from each feeding group. Though the male CONV group showed a slightly lower BIRC2 protein abundance than both the ISO control and GMO group, something not seen at the RNA level, neither the male nor female GMO-fed rats showed an increase compared to the ISO-fed rats, so that the transcript levels did not correlate with the protein results (Fig. 2C).

### Cluster Analysis of UPR pathway-associated genes reveals some grouping of the three feeding groups

The comparisons of the individual RNAs between the feeding groups did not show any apparent pattern that would indicate a change in the associated pathways. To confirm this result, we further analysed our data by un-supervised hierarchical clustering analysis of the ΔCt values as this was expected to reveal any concerted changes of the pathways even if individual changes did not reach statistical significance.

**Figure 3:**
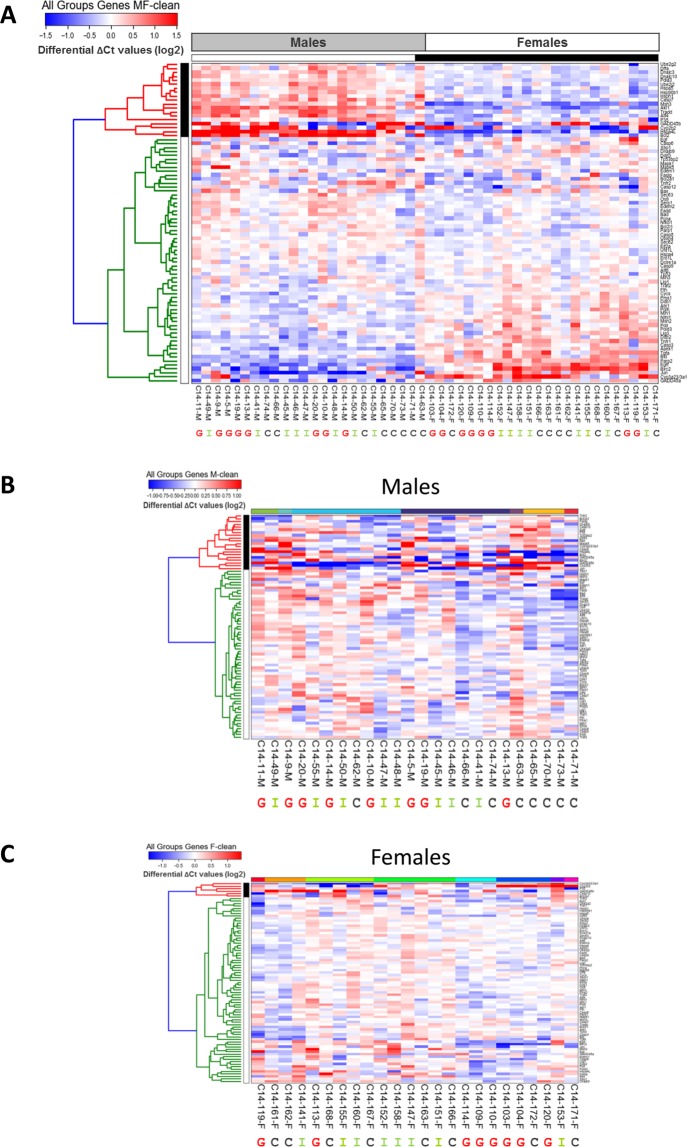
Cluster analysis of the samples based on ΔCt values obtained by RT-qPCR. Heat-map showing the relative abundance of 81 RNAs associated with apoptosis, NF-кB -, DDR-, and UPR pathways in the individual rats based on ΔCt-values obtained by qPCR. HOPCH clustering was performed on the whole cohort (**A**), as well as on the individual male (**B**) and female cohort (**C**). ΔCt values were normalised to the median for each row and colours reflects the variation from the median (log2). Individual samples are highlighted below the heat-maps as belonging to either the GMO (**G**), ISO (**I**) or CONV (**C**) feeding groups. Sample clusters are shown by black and white or coloured bars at the top.

First, we analysed both male and female cohorts together to see whether there were common changes that would lead to the individual rats to be clustered according to their feeding group. This analysis revealed two major clusters of male and female rats, showing that the most prominent difference between the animals was in fact not between feeding groups but the sexes, with only one male rat of the conventional feed cohort having been included in the ‘female’ cluster. The feeding groups themselves did not group together in either cluster (Fig. 3A).

As the major difference was found to be between the two sexes, we next analysed the two groups separately to identify any possible sex-related differences in the response to the feed. Again, the analysis of the male cohort did not show a clear separation of the feeding groups. Though five out of the eight rats of the CONV group clustered side by side they were separated into three individual clusters, while the other rats were spread over four further clusters. Similar results were observed for the female cohort, where five rats from the GMO group were side by side, but in two separate clusters with the overall cohort forming eight clusters (Fig. 3B).

**Figure 4:**
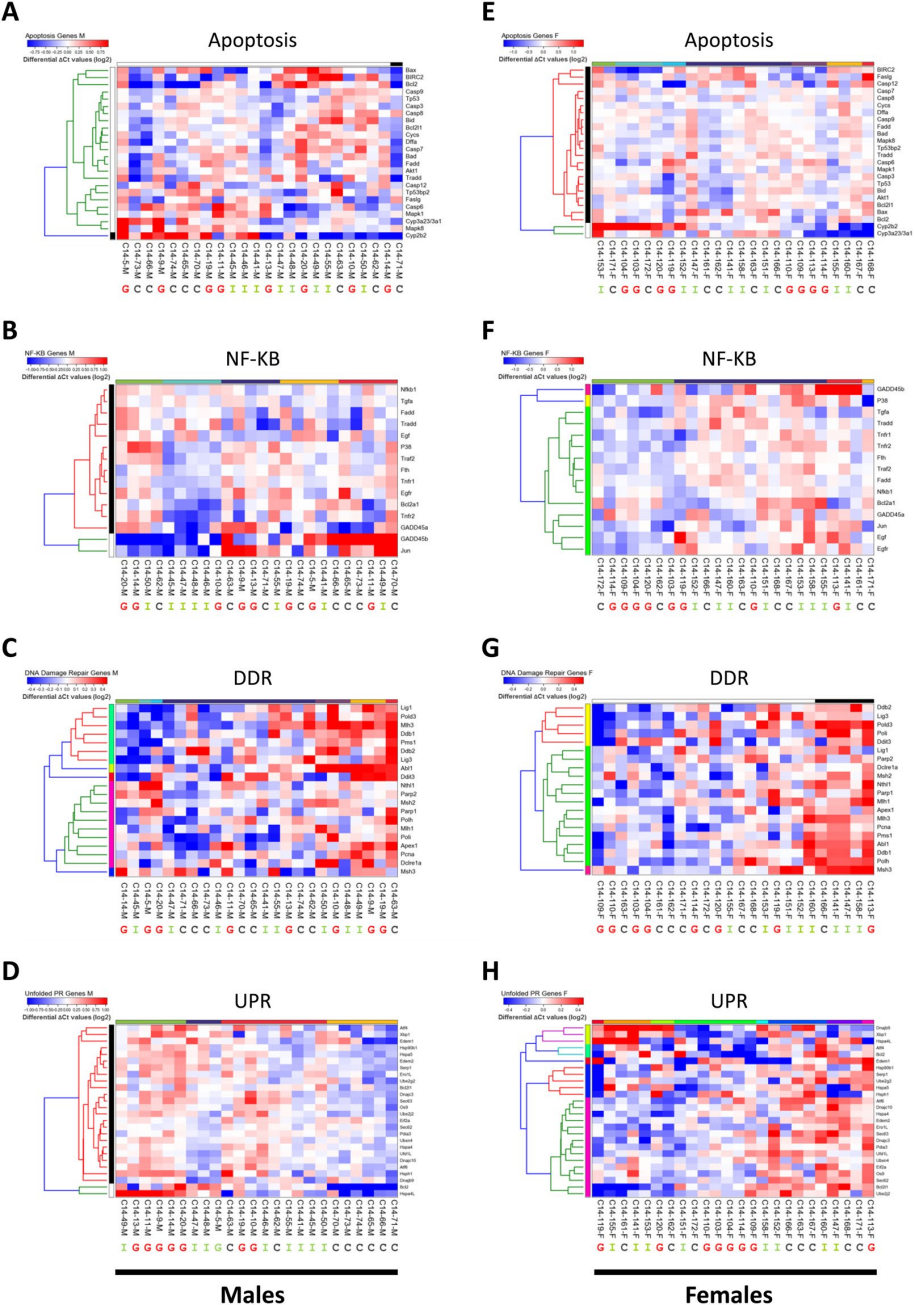
Cluster analysis of the samples based on ΔCt values of individual biological pathways and sex. Heat-maps of RNAs associated with apoptosis (24 RNAs), NF-кB - (15), DDR- (20), and UPR (26) pathways in the male (**A–D**), and female cohort (**E–H**). ΔCt values were again normalised to the median for each row and colours reflects the variation from the median (log2). Individual samples are highlighted below the heat-maps as belonging to either the GMO (**G**), ISO (**I**) or CONV (**C**) feeding groups. Sample clusters are shown by black & white or coloured bars at the top of each heat-map.

Though the unsupervised cluster analysis did not show any significant clustering of the feeding groups, it was still conceivable that individual pathways were changed. It was expected that this should become visible when the RNAs associated with the individual pathways were analysed as separate groups. The rats were therefore separately clustered depending on either the apoptosis-, DDR-, NF-кB -, and UPR-related pathway RNA abundance. Neither the male nor female cohorts showed any clear clustering of the feeding groups when apoptosis-, DDR- or NF-кB pathways were assessed (Fig. 4A–C,E–G). However, the UPR pathway-associated RNAs of the female cohort did show some clustering of the feeding groups, with one cluster including five of the eight GMO-fed rats, which also included one rat from the ISO and one from the CONV group (Fig. 4H). The other three GMO-fed rats were spread out evenly. The male cohort showed one cluster including six CONV-fed rats as well as one cluster with five GMO-fed rats and one ISO-fed rat. All others were spread over two further clusters (Fig. 4D).

It therefore appeared that the GMO and CONV feeding groups showed some differences in RNA expression of the UPR pathway. Indeed, when analysed as a group 14 out of 26 UPR-associated RNAs showed statistically significant decreases in the GMO group compared to the CONV group (Table S3). Notably, this difference was not found between the GMO and ISO group, or indeed between the ISO and CONV group. Further, whereas the male GMO appeared to show an overall slightly reduced level of RNA abundance compared to the CONV group, this was not the case in female rats (Fig. 4H; Table S3).

In summary, we did not detect consistent significant alterations in rat liver gene expression that would indicate an adverse effect after long-term feeding of MON810 compared to feeding with isogenic control maize. The largest variability observed was between the sexes rather than the feeding groups. Nevertheless, a difference in abundance of UPR RNAs of the male GMO and CONV feeding groups was observed, something not seen between the GMO and ISO-control group or in the female cohort.

## Discussion

GM crops have become a major part of animal feed in countries world-wide, and though no clear evidence has so far been presented that has shown a particular health hazard of these crops, safety concerns have repeatedly been raised^13,15^. It is hence important for any food and feed obtained from GM crops to be thoroughly assessed for its safety in order to make sure that no unintended changes through e.g. animal feeding trails could occur^7^. In 2012, the “Guidance for the risk assessment of food and feed derived from GM animals and related animal health and welfare aspects” was therefore published by the European Food Safety Authority (EFSA)^23^. This described guidelines on how to properly assess the risk comparing GM food/feed with their respective conventional counterparts. In their guidelines, the concept of substantial equivalence was proposed to evaluate the similarities and differences between GM foods and their conventional counterparts^23^. Testing the substantial equivalence between the GM plants and the unmodified parent strain was an integral part of the safety evaluation and should be the starting point for the overall assessment.

With this in mind and following those guidelines, we previously performed a study of a one-year feeding regime with conventional maize containing either 33% GMO maize MON810, non-GM isogenic control maize or only conventional maize in their diets, and these data have recently been published^22^. In this study, we assessed the weight and possible histological changes which would be expected if MON810 long-term feed was to have any effect on the animals’ health. However, none of the variables tested showed any significant alteration that could be contributed to the MON810 feed itself. The main aim of the current study following on from these findings was to further evaluate selected marker genes of key biological pathways using targeted RT-qPCR.

Because of the previous findings by de Vendemois *et al*.^15^ of potential hepatotoxic effects, as well as the importance of the biological pathways mentioned above, we therefore focussed our research on the liver and in particular on the expression of genes associated with the major stress pathways. Both, the apoptosis and NF-кB pathways are key pathways affecting cell survival and controlling proliferation, and have been shown to be involved in many diseases, including pathologies of the liver^24^. Evidence shows that hepatocyte apoptosis contributes to a number of liver diseases, including alcohol-induced liver disease, viral hepatitis, cholestatic liver disease, non-alcoholic fatty liver disease, and Wilson’s disease^25^. DDR signalling is another fundamental pathway affecting human and animal diseases, such as liver cancer^26^, and which has been found activated in rodent liver upon feeding with cell-damaging agents^27–29^. The unfolded protein response pathway is also activated upon cellular stress or exposure to certain drugs^30^, and is related to an accumulation of unfolded or wrongly folded proteins within the endoplasmic reticulum (ER)^31^. It is activated in many diseases^32,33^ but can also be activated through obesity, through a high fat-diet, and multiple other stress factors^34,35^.

Consistent with the results from our previous study, our data did not describe a gene expression pattern consistent with any short or long term adverse effect. With the exception of *Birc2*, none of the RNAs showed consistent changes in abundance of the non-stringent cut-off of ≥1.5-fold in both male and female rats. Despite the increase in *Birc2* RNA, this did not translate into a corresponding protein increase. Because of this result, and as we could not find any other corresponding significant transcript changes that reflect the changes seen for Birc2 in a positive or negative way (Figs. 3B,C & 4A,E**)**, it was unlikely that this difference was indeed biologically relevant.

RT-qPCR is the recognised highly sensitive method of choice to quantify any potential transcriptional changes that would reflect biological alterations. Although our selection of 86 RNAs may not have included all possible RNAs associated with the indicated pathways, as e.g. full-genome transcriptome analysis would have done, we would have strongly expected to see large variations in the majority of the RNAs encoding proteins of the same biological pathway, which would also translate into changes in protein expression if those pathways had been significantly affected. However, this was not observed. Whether the change in *Birc2* RNA abundance reflects a biological or technical variation is unknown. However, it is not unusual for RNA levels to not directly correlate with protein levels because of posttranscriptional alterations and protein level control mechanisms that could counteract changes in RNA abundance. In addition, the chances that statistically significant changes of individual RNAs are identified in such a study, especially for low abundance RNAs, are relatively high with this very sensitive technology, as even very small variations in sample handling can affect the final result to a much larger degree or can depict e.g. circadian effects^20^. In addition, naturally occurring biological variation of individuals also always needs to be taken into account. In fact, when four randomly chosen male GMO-fed rats were compared to the other four of the same feeding group, differences of ≥1.5-fold were also observed (data not shown). A statistically significant change of one RNA is therefore not sufficient evidence that a particular cellular or physiological pathway has been affected. In addition, statistically significant changes were found in all six comparisons, including the comparison of isogenic non-GM maize to a conventional maize.

Though direct comparison of individual genes identifies the differential expression of genes encoding key proteins of a pathway, statistically significant changes of individual RNAs will always occur randomly without the overall pathway being affected. Another approach was therefore to look for concerted changes in expression of genes related to a specific pathway and/or physiological process. We used the unsupervised hierarchical clustering tool within ‘Altanalyze’ software, which allowed us to cluster the individual rats by HOPACH clustering^36^ to look for concerted RNA changes associated with individual feeding groups. This showed that the strongest discriminator was in fact the sex of the rat and not the feeding group they belonged to. Within the male and female cohort no apparent clustering of the feeding groups was observed when all 81 genes were included, showing that any potential differences were well below the natural differences found between sexes. We could also not see any evidence for an apoptotic, DDR, or NF-кB activation, which would have indicated a cell damage or inflammatory response. Only the RNA expression patterns of the UPR pathway, which is activated upon a cell’s stress within the endoplasmic reticulum (ER stress), did lead to some clustering of the male feeding groups. Indeed, 14 out the 26 UPR-associated RNAs showed a significant difference between the GMO and CONV groups, even though the vast majority of these changes was very low and well below the chosen 1.5-fold cut-off. However, it is notable that this difference was not seen between the GMO and ISO group, which would indicate that the Bt-transgene itself did not have a significant impact. Neither was it observed between the ISO and the CONV group, so that we currently do not have an explanation for our finding. Interestingly, the male GMO group showed the lowest expression (highest ΔCt values) followed by the ISO group, and the CONV-fed group showing the highest relative expression. The UPR responds to an accumulation of unfolded proteins within the ER^37^, and upon prolonged high expression can induce cell death, though low expression can in fact improve cell survival^31,38^. A low level expression appears to be present in most cells, where it is involved in quality control of protein folding^39^. Further activation in the liver has been found e.g. upon poorer nutrition, e.g. through a high fat/low protein diet^37,40^, obesity and type 2 diabetes^41^. We do not know whether this does indeed reflect a reduced ER-stress within the GMO-fed rats’ liver as this has not been tested any further. The different feeds used in this study had been chosen as they have highly similar nutrient contents, so that it is unlikely that a difference based on the nutritional values has affected the study. Further, variations in the expression of stress-related proteins have recently been described in a proteomic analysis of the rat small intestine after a 7-day and 28-day feeding regime with 33% corn; however, this occurred when rats were fed transgenic (including MON810) or non-transgenic corn varieties and could therefore not be attributed to the transgenic corn diet^42^. Therefore, the underlying reason for our finding still remains to be elucidated. Finally, our distinct liver mRNA results are consistent with our previous study data, in which no differences in overall weight or body condition and no abnormal histological liver findings were observed between the feeding groups^22^.

## Conclusion

In summary, our results underline that long-term feeding of MON810 maize does not trigger physiological changes in rat liver in a 1-year feeding approach based on the expression of distinct members of the DNA damage, inflammation or cell death response. Some effect on the liver transcriptome of the UPR pathway stress-response genes were found in male rats, but these changes could not be directly attributed to the expression of the transgene itself. Therefore, our results do not describe a consistent biological response in male and female rats that could be clearly attributed to the MON810 feeding programme compared to the two control feeding groups.

## Materials and Methods

### Rat feeding trials

The rat feeding trial was conducted as part of the GRACE project (“GMO Risk Assessment and Communication of Evidence”) in accordance with the relevant national legislation on the use of animals for research and has been described previously^22^. It was performed in compliance with Good Laboratory Practice in the animal house at the Department of Toxicology of the Slovak Medical University, Bratislava (Slovakia), and had been approved by the local ethics committee. OECD TG 452 guidelines (OECD 2009) and recommendations included in the EFSA Guidance on conducting repeated-dose oral toxicity studies in rodents on whole food/feed (EFSA Scientific Committee 2011) have been taken into account. Diet preparation and animal study design have been described in that publication. In brief, per group and gender 20 male and 20 female Wistar Han RCC rats with a uniform weight were randomly assigned to either the GMO, isogenic control or conventional treatment group (120 animals in total). The GMO group contained 33% MON810 maize, the isogenic control group 33% near-isogenic non-GM maize and the conventional group was fed a diet including 33% conventional maize. Eight rats from each group of 20 were randomly selected for analysis. Thus, 24 male and 24 female rats were analysed in total. Liver tissue was dissected after sacrifice, immediately frozen in liquid nitrogen, and stored at −80 °C until further use.

### RNA extraction and reverse transcription

250 mg wet-weight tissue samples were used to isolate total RNA using the mirVana™ miRNA Isolation Kit (Ambion) according to the manufacturer’s protocol for total RNA. Yield was quantified at 260 nm and purity assessed by 260/280 nm ratio using the Nanodrop 1000 Spectrophotometer (Thermo). Further quality assessment was carried out as described previously using the 2100 Bioanalyzer (RIN > 8, Agilent Technologies)^43^. All samples were stored at −80 °C until further use.

To remove genomic DNA DNase treatment of the isolated RNA was performed before reverse transcription (Thermo Scientific) according to manufacturer’s protocol. For first strand cDNA synthesis, 1 µg of total RNA was reverse-transcribed using the RevertAid™ M-MuLV Reverse Transcriptase (Fermentas GmbH) in a reaction volume of 20 µl following the manufacturer’s instructions. The final cDNA was stored in aliquots at −20 °C until further analysis.

### Quantitative real-time PCR (qPCR)

Quantification of mRNA expression was performed by RT-qPCR as described previously^44^, with some modifications. In brief, SYBR Green qPCR was performed using the SensiMix DNA Kit (Quantace Ltd.). 0.2 µM of gene specific primers were added per reaction (Table S1). All oligonucleotides were synthesized by Sigma Aldrich. Amplification was carried out under the following conditions: denaturation at 95 °C for 2 min, followed by 40 cycles with 15 s at 95 °C, 10 s at 60 °C and 10 s at 72 °C. Melting curve analysis was used to confirm product specificity of each qRT-PCR reaction. All primers were optimized by serial dilution of PCR products to detect efficiency, and the primers with efficiency of at least 80% were used for all reactions. All amplicons were verified by sequencing. Reactions were carried out in triplicate using 1 µl of 1:5 diluted cDNA in a 10 µl final reaction volume in a PikoReal™ Real-Time PCR System (Thermo Scientific). Normalisation of expression was performed using three stably expressed reference genes (B2M, HPRT1 and RPLP1). Relative gene expression was obtained by calculating the ∆∆Ct values between the GMO group, isogenic group and the conventional group.

### Statistical analysis

All data was expressed as mean ΔCt ± standard deviation (SD) from eight samples for each group and processed using Excel (Table S2). Two-sided student T-Tests were used to test for significance of difference of the means. P ≤ 0.05 was considered statistically significant.

Unsupervised hierarchical (HOPACH) clustering of individual samples was performed and visualised using the default settings of the open-source ALTANALYZE software (http://www.AltAnalyze.org)^45^ based on the ΔCt values with in-row normalisation to the respective median.

### Western blot

Total liver protein was extracted using RIPA buffer and quantified using a BCA assay. Equal amounts of the protein extracts from the individual samples were pooled and 20 μg of total protein per sample were separated on a 10% (w/v) Tris-Glycine SDS-PAGE gel and subsequently transferred onto a 0.45 µm nitrocellulose blotting membrane (Sartorius) by semi-dry electro-blotting for 60–80 min at 1.2 mA/cm^2^. The membrane background was blocked with 5% skimmed milk (Carl Roth) in TBST for 1 h at room temperature, followed by incubation with primary antibody (BIRC2 (#4952; 1:1,000), GAPDH (#5174; 1:1,000); all Cell Signaling) at 4 °C overnight. Membranes were washed three times in PBST for 10 min and then incubated with the appropriate horseradish-peroxidase linked secondary antibody for 1 h at room temperature. Immuno-reactive proteins were visualized using enhanced chemi-luminescence (ECL Select, Amersham) and the documentation system Fusion SL (VilberLourmat).

## Supplementary information

Table S1

Table S2

Table S3
